# Myosin II-Mediated Focal Adhesion Maturation Is Tension Insensitive

**DOI:** 10.1371/journal.pone.0070652

**Published:** 2013-07-29

**Authors:** Jonathan Stricker, Yvonne Beckham, Michael W. Davidson, Margaret L. Gardel

**Affiliations:** 1 Institute for Biophysical Dynamics, James Franck Institute, University of Chicago, Chicago, Illinois, United States of America; 2 Department of Physics, University of Chicago, Chicago, Illinois, United States of America; 3 National High Magnetic Field Laboratory, Florida State University, Tallahassee, Florida, United States of America; University of California, Berkeley, United States of America

## Abstract

Myosin II motors drive changes in focal adhesion morphology and composition in a “maturation process” that is crucial for regulating adhesion dynamics and signaling guiding cell adhesion, migration and fate. The underlying mechanisms of maturation, however, have been obscured by the intermingled effects of myosin II on lamellar actin architecture, dynamics and force transmission. Here, we show that focal adhesion growth rate stays constant even when cellular tension is reduced by 75%. Focal adhesion growth halts only when myosin stresses are sufficiently low to impair actin retrograde flow. Focal adhesion lifetime is reduced at low levels of cellular tension, but adhesion stability can be rescued at low levels of force by over-expression of α-actinin or constitutively active Dia1. Our work identifies a minimal myosin activity threshold that is necessary to drive lamellar actin retrograde flow is sufficient to permit focal adhesion elongation. Above this nominal threshold, myosin-mediated actin organization and dynamics regulate focal adhesion growth and stability in a force-insensitive fashion.

## Introduction

Cell adhesion to the extracellular matrix (ECM) is dynamically regulated during directed cell migration, tissue morphogenesis and wound healing [1], [2]. As sites of attachment between the cell and ECM, focal adhesions (FA) play an important role in regulating cell morphology, cell-ECM force transmission and ECM-mediated signaling [3]. Adhesions assemble in an actin polymerization-dependent manner within the lamellipodium and stabilize to the ECM near the lamellipodial base [1], [4], [5]. Myosin II motors within the lamella drive changes in focal adhesion morphology and composition in a “maturation process” that is crucial for regulating focal adhesion dynamics and mechanotransduction pathways [6].

Understanding the mechanisms of myosin-mediated focal adhesion maturation has been complicated by the intermingled effects of myosin II on lamellar actin architecture, dynamics and force transmission at the focal adhesion plaque [1], [4], [7]. Stresses generated by myosin II drive retrograde flow of the lamellar actin cytoskeleton and are transmitted to focal adhesion proteins, which are thought to respond in a force-dependent manner. Myosin-mediated cross-linking also facilitates reorganization of lamellar actin in a formin (Dia1) and α-actinin dependent manner to form a dense radial stress fiber (RSF) at the focal adhesion [4], [7], [8], [9]. While it is widely presumed that focal adhesion maturation is a tension-dependent process [2], [8], [10], [11], several recent studies have implicated the importance of myosin II cross-linking and radial stress fiber assembly in focal adhesion growth [4], [7]. Thus, the extent to which mechanical tension is a direct regulator of focal adhesion growth and stabilization is not well established.

Here, we demonstrate that myosin II-mediated focal adhesion growth and stability is, to a large extent, a tension-independent process and instead is regulated by myosin II-mediated actin retrograde flow and bundling. Using the Rho-kinase inhibitor, Y-27632, we modulated the extent of myosin II activity by varying the inhibitor concentration and measured the changes in cellular tension using traction force microscopy. We found that mature focal adhesions persisted even when cellular tension was reduced by 75% and were abrogated only after complete myosin inhibition, reflecting a reduction in cellular tension of 90%. Using live cell imaging to track focal adhesion dynamics and actin retrograde flow, we found that both the focal adhesion growth rate and actin retrograde flow rate stayed remarkably constant as tension was reduced by 75%. The focal adhesion lifetime was reduced upon myosin II inhibition, but could be recovered by over-expression of the actin cross-linking protein α-actinin or a constitutively active form of the nucleation promoting factor Dia1. Our work identifies a minimal threshold of myosin activity required to drive actin retrograde flow is necessary for adhesion growth. However, above that tension threshold, actin retrograde flow dynamics and architecture are more crucial determinates of focal adhesion growth rate and lifetime, respectively.

## Results

### Focal adhesion elongation occurs along an actin bundle template

**Figure 1:**
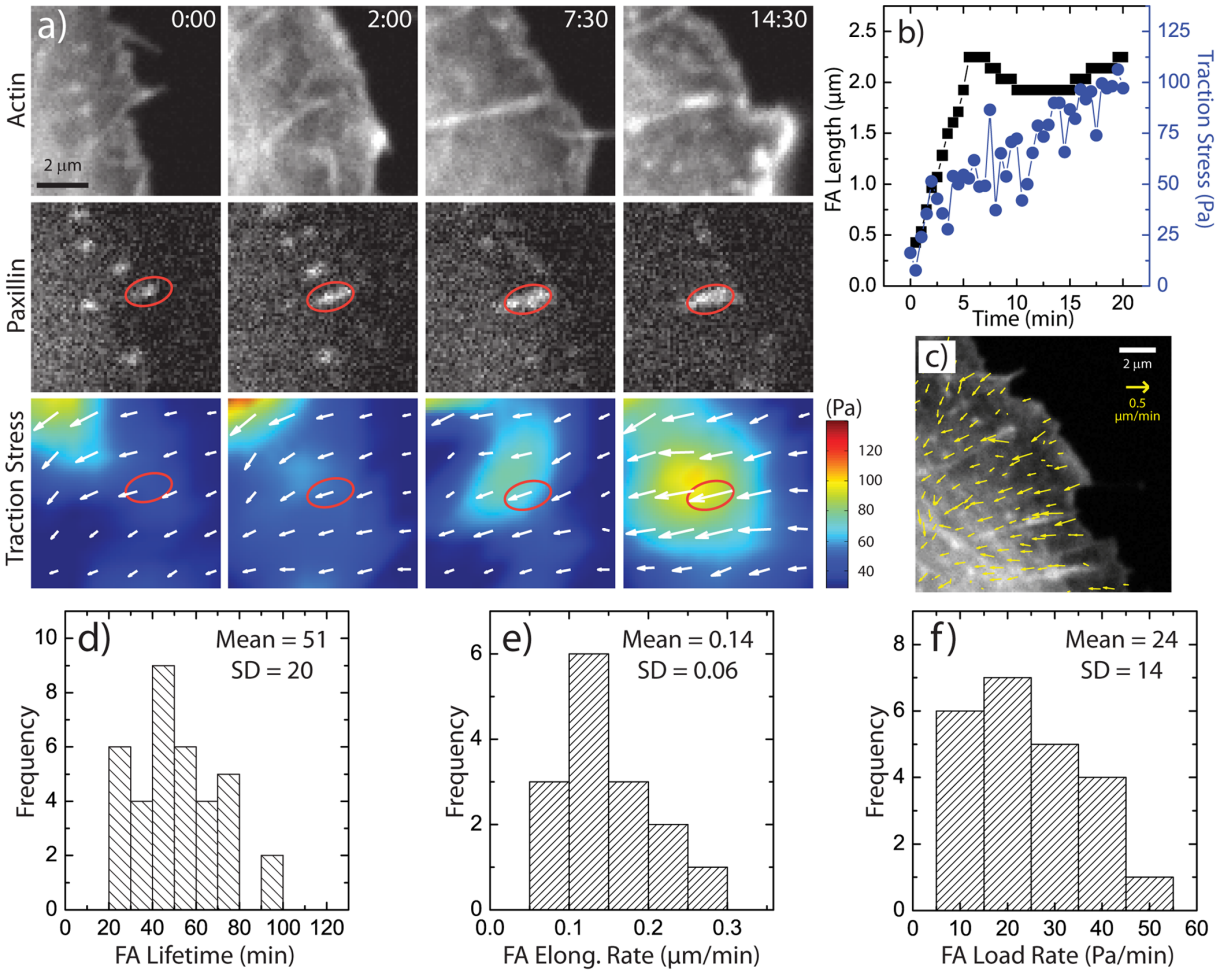
Focal Adhesion Maturation in Wild Type Cells. (a) Time-lapse images of a U2OS cell expressing GFP-actin and mApple-paxillin, with images of traction stress magnitude and overlaid traction stress vectors. Time is in min:sec. (b) Plot of focal adhesion length (black squares) and traction stress (blue circles) at the focal adhesion (FA) indicated in (a) as a function of time. (c) Region of a U2OS cell with overlaid actin retrograde flow vectors. (d) Histogram of FA lifetimes in U2OS cells. (e) Histogram of FA elongation rate, measured by the rate of change in the length during FA elongation. (f) Histogram of focal adhesion loading rate, measured by the rate of change of the traction stress during FA elongation.

U2OS human osteosarcoma cells were transfected with GFP-actin and mApple-Paxillin and plated on fibronectin-coated traction force substrates compatible with high resolution imaging (Video S1). Consistent with previous reports [4], [7], nascent focal adhesions underwent elongation into mature focal adhesions concomitantly with the assembly of a radial stress fiber (Fig. 1a+b). During this process, a constant retrograde flow of actin persisted across the lamella at a rate of 0.3 µm/min (Fig. 1c), constituting a “slipping clutch” between lamellar actin and the focal adhesion plaque [5], [12], [13]. During these changes to focal adhesion size and actin organization, the traction stress exerted on the underlying substratum increased from background levels to nearly 100****Pa.

Time-lapse imaging of GFP-paxillin over 30–60 min enabled measurements of focal adhesion dynamics. Focal adhesions which elongated into mature focal adhesions persisted for an average of approximately 55 minutes before disassembling, characterizing a focal adhesion lifetime (Fig. 1d). The rate of FA growth during maturation, or its “elongation rate” was peaked around 0.15 µm/min (Fig. 1e). The rate at which tension increased at the focal adhesion plaque, or “loading rate”, was broadly peaked around a mean of 25 Pa/min (Fig. 1f). Thus, consistent with previous measurements we observed a correlation between tension and focal adhesion size during assembly [14], [15], which has previously been interpreted as evidence for focal adhesion mechanosensitivity [8].

### Adhesion maturation persists when cellular tension is reduced by 75%

**Figure 2:**
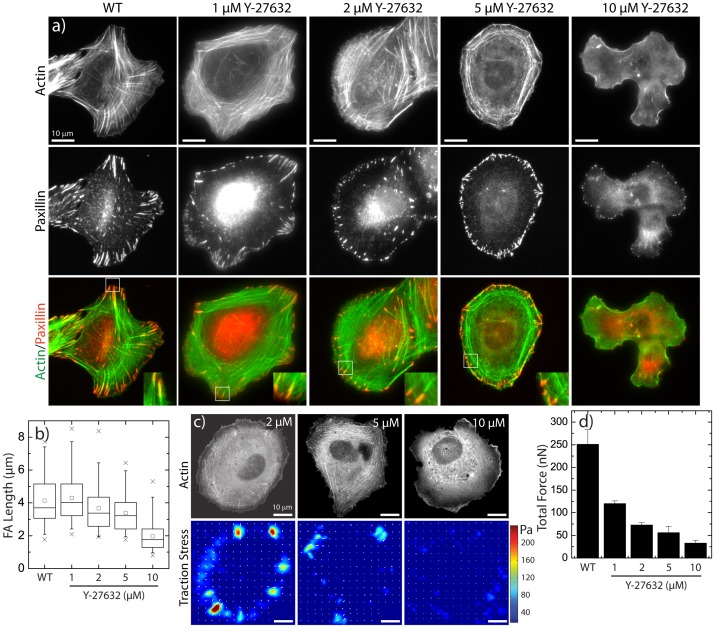
Focal Adhesion Morphology and Traction Forces under Treatment with ROCK inhibitor Y-27632. (a) Images of F-actin visualized by fluorescent phalloidin (top row) and paxillin immunofluorescence (middle row) in U2OS cells treated with the indicated concentrations of the rho-kinase inhibitor, Y-27632. (b) Box plots of FA lengths in U2OS cells as a function of Y-27632 concentration (n = 101 (WT), 78 (1 µM), 134 (2 µM), 117 (5 µM), 124 (10 µM) FAs). (c) Representative GFP-actin images (top row) and traction stress maps (bottom row) and for U2OS cells treated with 2, 5 and 10 µM Y-27632. (d) Plot of the total traction force exerted by U2OS cells as a function of Y-27632 concentration (n = 23 (WT), 13 (1 µM), 8 (2 µM), 14 (5 µM), 6 (10 µM) cells).

In order to assess the role of tension in the focal adhesion elongation process, we modulated myosin activity by incubating the cells with varying concentrations (1–10 µM) of the Rho-associated protein kinase (ROCK) inhibitor Y-27632 for 1 hour prior to fixing and staining for actin and paxillin. As expected, at high concentrations (10 µM) of Y-27632, lamellar actin bundles were completely abrogated and focal adhesions appeared as near diffraction-limited puncta near the cell edge (Fig. 2a+b). However, at low concentrations of the inhibitor (2 µM), transverse arcs and radial stress fibers terminating in elongated focal adhesions were still observed (Fig. 2a+b). As the concentration was increased to 5 µM, the appearance of radial stress fibers was markedly reduced but some elongated focal adhesions terminating in radial stress fibers still remained (Fig. 2a+b). Thus, elongated focal adhesions persisted at intermediate concentrations of Y-27632 (Fig. 2b).

To assess the cellular tension at each of these drug concentrations, we summed the magnitude of the traction stress over the entire cell area to obtain the total traction force. Control cells exerted approximately 250 nN of traction force (Fig. 2c+d). Upon treatment with 1 µM Y-27632, the traction force was reduced by ∼50% and continued to decrease further as the concentration was increased (Fig. 2c+d). In 10 µM Y-27632, the traction force exerted was reduced to 25 nN, approximately 10% that of control cells and consistent with previously reported values of the myosin-independent force exerted by adherent cells (Fig. 2c+d) [16], [17]. Thus, despite reductions in cellular tension by 50–80% in intermediate concentrations of Y-27632 (Fig. 2b+c), elongated focal adhesions persisted.

### Reduced myosin activity shortens FA lifetime but does not affect their growth rate

**Figure 3:**
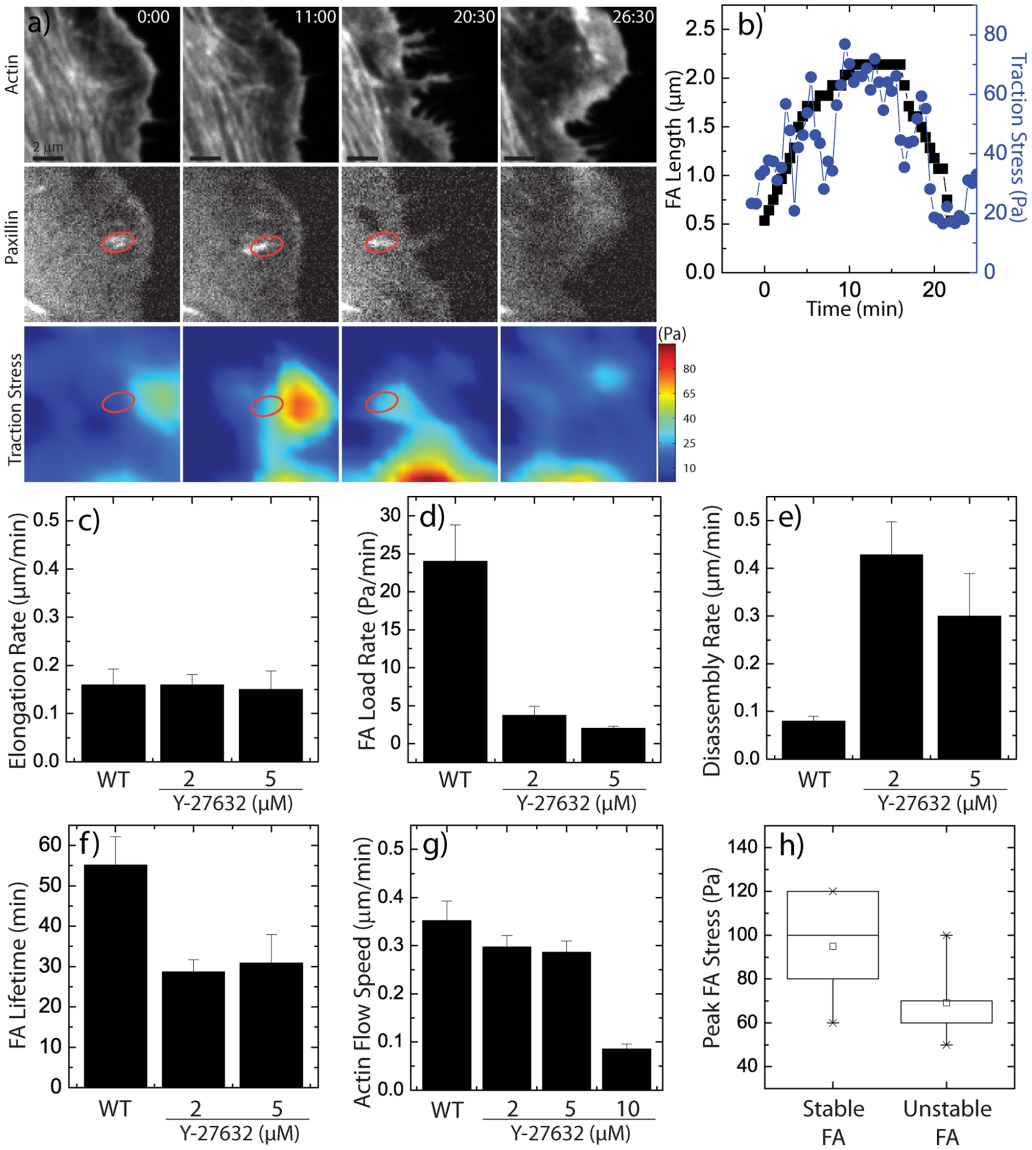
Focal Adhesion Maturation under Treatment with ROCK inhibitor Y-27632. (a) Time-lapse images of GFP-actin and mApple-paxillin in U2OS cell treated with 2 µM Y-27632, with images of traction stress magnitude. Time is in min:sec. (b) Focal adhesion length (black squares) and traction stress (blue circles) at the focal adhesion indicated in (a) as a function of time. Focal adhesion assembly characteristics in varied Y-27632 concentrations: (c) FA elongation rate, (d) FA loading rate, (e) FA disassembly rate, (f) FA lifetime under indicated conditions (n = 10 FA for each condition for FA elongation, loading rate, disassembly rate, and lifetime). (g) Lamellar actin retrograde flow speeds in varied conditions (n = 2 cells per condition, 5 to 7 regions per cell). (h) Box plot of peak traction stress reached during focal adhesion lifetimes in cells treated with Y-27632. “Stable FA” indicates FA that had lifetime greater than 30 minutes, while “Unstable FA” indicates FA with lifetime less than 30 minutes.

To assess changes in focal adhesion dynamics at intermediate levels of Y-27632 where elongated focal adhesions were observed despite significantly reduced cellular tension, we imaged GFP-actin and mApple-paxillin in drug-treated cells plated on traction force substrata (Video S2–S4). For cells treated with 2 and 5 µM Y-27632, focal adhesion assembly appeared remarkably similar to that of control cells. Nascent focal adhesion puncta appeared at the cell edge and elongated as traction stress increased (Fig. 3a+b). Surprisingly, the FA elongation rate was unaffected (Fig. 3c) despite a dramatic decrease in the loading rate (Fig. 3d). However, an enhanced disassembly rate (Fig. 3e) resulted in a significantly reduced focal adhesion lifetime (Fig. 3f).

The constant focal adhesion elongation rate was surprising because of the dramatically reduced loading rate. If tension were an important parameter in focal adhesion elongation, we would have expected a corresponding decrease with reduced cellular tension. Two other roles of myosin activity that may persist at these intermediate tensions are myosin-driven retrograde flow and cross-linking. Indeed, we found that actin retrograde flow persisted at a constant rate in cells treated with 2 and 5 µM Y-27632 and was only abrogated at the highest concentration of 10 µM Y-27632 (Fig. 3g). Interestingly the speed of retrograde flow (0.3 µm/min) was approximately twice that of FA elongation (0.15 µm/min), suggesting that constant FA retrograde flow dynamics control the elongation rate of FA over a broad range of cellular tension.

At these reduced levels of myosin activity, focal adhesion stability was severely impacted, with lifetimes being reduced by nearly 50% (Fig. 3f). Interestingly, under partial myosin inhibition, we observed a fraction of adhesions which elongated but which were unstable, having a lifetime of less than 25 min. The maximal traction stress generated at these unstable focal adhesions remained low, approximately 60 Pa (1.8 nN) on average. By contrast, the population of stable FAs that elongated and then persisted for at least 30 minutes typically built up a tension of 100 Pa (3 nN) (Fig. 3h).

### Over-expression of α-actinin or Dia1 stabilizes FAs under low tension

**Figure 4:**
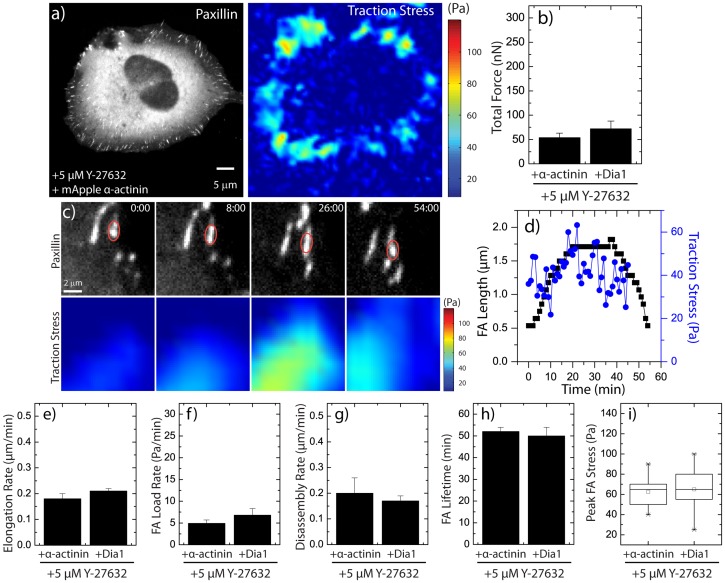
Focal Adhesion Stability is Rescued at Low Tension by Over-expression of α-actinin and Dia1. (a) Representative GFP-paxillin image and traction stress map of a U2OS cell treated with 5 µM Y-27632 and over-expressing mApple-α-actinin. (b) Total traction force generated by cells treated with 5 µM Y-27632 and over-expressing either mApple-α-actinin or mCherry-Dia1 (n = 9 cells per condition). (c) Time-lapse GFP-paxillin and traction stress map images of a U2OS cell treated with 5 µM Y-27632 and over-expressing mApple-α-actinin. Time is in min:sec. (d) Focal adhesion length (black squares) and traction stress (blue circles) at the focal adhesion as a function of time for red oval indicated in (c). Focal adhesion assembly characteristics for cells treated with 5 µM Y-27532 and over-expressing either mApple-α-actinin or mCherry-CA-Dia1: (e) FA elongation rate, (f) FA loading rate, (g) FA disassembly rate, (h) FA lifetime and (i) Maximum FA stress attained by FAs (n = 12 FA for each condition for FA elongation, loading rate, disassembly rate, and lifetime).

Our previous work showed that a stress fiber template is necessary for focal adhesion maturation (Oakes et al., 2012). In order to assess the role of radial stress fibers in stabilizing and elongating focal adhesions at low tensions, we chose to explore the roles of two proteins known to play important roles in the formation of RSFs: the actin cross-linker α-actinin 1, and the actin nucleator Dia1 [7], [9], [17]. Constructs of mApple α-actinin or constitutively active Dia1 were over-expressed in U2OS cells treated with 5 µM Y-27632, and traction forces and adhesion dynamics were obtained as previously described (Fig. 4a–d; Video S5+S6). For both constructs, cells exerted a total average force of between 50 to 75 nN (Fig. 4b), a level comparable with control cells treated with 5 µM Y-27632 (Fig. 2d).

The reduced level of cellular tension resulted in a loading rate during focal adhesion growth of 5 Pa/min (Fig. 4f), comparable to control cells treated with 5****µM Y-27632 (Fig. 3c,d) but at a similar elongation rate to all previous conditions (Fig. 4e). Over-expression of either α-actinin or CA-Dia1, however, resulted in a reduced disassembly rate (Fig. 4g) and an increased focal adhesion lifetime (Fig. 4h) comparable to control cells at normal levels of intracellular tension (Fig. 3e,f). Thus, promotion of RSF assembly was sufficient to stabilize focal adhesions at low levels of intracellular tension (Fig. 4i). This result suggests that an important role of myosin II activity in the stabilization of focal adhesions is in its function to assemble RSFs at focal adhesion sites.

## Discussion

Here we have demonstrated that a nominal amount of myosin-generated tension required to drive actin retrograde flow was sufficient to drive focal adhesion elongation. Promoting the assembly of a radial stress fiber at the focal adhesion plaque by α-actinin-mediated cross-linking or enhanced Dia1-mediated actin polymerization was sufficient to stabilize focal adhesion plaques under a large range of intracellular tensions. Thus, we have shown mechanical force *per se* is not a crucial parameter in regulating focal adhesion size or lifetime. Rather, a small level of myosin II activity is necessary to drive retrograde flow dynamics and to facilitate the assembly of radial stress fibers at focal adhesions.

The mechanism by which myosin II mediates focal adhesion maturation has long been presumed to be based on tension [8], [10], [14]. This assumption has led to the idea that focal adhesion plaques are mechanosensitive organelles, with a tension-dependent size and composition. However, the contributions of myosin-mediated retrograde flow and cross-linking have not been fully appreciated in previous studies of focal adhesion maturation. We showed previously that myosin II-dependent stabilization of nascent adhesions occurs at a critical tension [18]. After adhesion stabilization we, and others, have observed a correlation between adhesion size and local tension during adhesion growth [14], [15]. However, this correlation varied widely between individual FAs and we demonstrate here that this correlation is not causal – changes in loading rate between 7 – 25 Pa/min (∼0.2–0.75 nN/min) have no effect on FA growth rate. We speculate that the previously reported behavior of adhesion growth related to external load (Riveline et al., 2001) may have arisen either from strain or stress-induced formation of a radial stress fiber, or stress fiber thickening, as has been reported along the stress fiber length [19], [20].

That actin retrograde flow maintained a constant value over a range of Y-27632 concentrations is intriguing and indicates variable coupling between the actin and focal adhesion plaque at different levels of stress. The focal adhesion growth rate also remained constant, and was approximately half the retrograde flow rate. Interestingly, we previously found this correlation between flow rate and adhesion growth rate under perturbations that enhanced retrograde flow [7]. In a model of simple friction, the force generated by myosin II is balanced by the adhesive drag *ηv* at the focal adhesion site, where *η* is the frictional coupling between the actin and focal adhesion and *v* is the retrograde flow speed. Since the force is directly measured at the focal adhesion site and diminishes while *v* stays constant, *η* would also decrease under decreased myosin activity in this model. Such a picture would suggest an intimate coupling between myosin-generated tension and force transmission within the adhesion plaque. Another explanation for this data is that myosin tension induces local remodeling of the actin cytoskeleton in a tension-dependent manner, such as been observed during the formation of stress fibers and within stress fibers [17], [19], [20]. Distinguishing between these regimes quantitatively will require further experimental and modeling work.

This work also supports the importance of lamellar architecture in the regulation of adhesion maturation. Our previous work had shown that impaired RSF assembly impaired focal adhesion maturation despite high tension levels [7]. Here we demonstrate that promoting RSF formation by over-expression of α-actinin or CA-Dia is sufficient to stabilize adhesions even at very low tension. The mechanism by which RSFs assemble at adhesions is not clear, as they do not form when cell spread area is confined or cells are plated on soft matrices [17]. We speculate that myosin may be directly cross-linking actin or facilitating a local tension-dependent remodeling of the actin cytoskeleton, as has been observed along stress fibers. As RSF assembly has now been shown to play an essential role in adhesion maturation, determining the regulation of their assembly is an important problem requiring future work.

In conclusion, these results delineate the roles of myosin II in focal adhesion elongation. While previous models have suggested that focal adhesion growth may arise by tension-dependent mechanosensing within the focal adhesion plaque, our work demonstrates that the rate and extent of adhesion elongation is determined by myosin-mediated actin retrograde flow and cross-linking in a tension-insensitive manner. This observation calls into question the extent to which tension-sensing events within the adhesion plaque dominate focal adhesion assembly and growth and suggests a dominant role of the actin cytoskeleton architecture and dynamics in environmental sensing and regulation of cell adhesion.

## Materials and Methods

### Cell culture

U2OS human osteosarcoma cells (ATCC) were cultured in McCoy's 5A media (Sigma) supplemented with 10% fetal bovine serum (HyClone), 2 mM L-glutamine (Gibco) and penicillin-streptomycin (Gibco). Cells were transfected with plasmid DNA constructs encoding for GFP-actin (gift of the Gary Borisy Lab, Northwestern University), mApple-paxillin and mApple-α-actinin, EGFP-paxillin [21], GFP-Dia1 (gift of the Henry Higgs lab) using the transfection reagent FuGENE 6 (Roche). Y-27632 was purchased from Calbiochem and used at concentrations of between 1 and 10 µM as indicated. Cells were plated for 24 hours and then incubated for 1 hour in Y-27632 at the concentration indicated for all experiments.

### Polyacrylamide substrates for traction force microscopy

Polyacrylamide (PAA) substrates containing far-red 40 nm fluorescent microbeads (Invitrogen) were prepared on glass coverslips using previously published methods [22], [23]. Briefly, PAA gels with 7.5%/0.1% weight percentage of acrylamide/bis-acrylamide were used to create a gel with a shear elastic moduli of 2.8 kPa [22], [24]. Fibronectin (Millipore) was coupled to the surface of the PAA gels by means of hydrazine hydrate [15], [22], [25], as previously described. Briefly, PAA gels were incubated for at least 2 hours in undiluted hydrazine, followed by a 1 hour incubation in 5% acetic acid and then washed. A 10 µg/mL fibronectin solution was prepared in sodium acetate buffer (pH 4.5), and oxidized by addition of 40 µg/mL sodium meta-periodate prior to a 30 min incubation on the PAA gel at room temperature. The PAA gels were then rinsed repeatedly and plated with cells.

### Microscopy

Live cell traction force measurements were performed on an inverted Nikon Ti-E microscope with a CSU-X confocal scanhead (Yokogawa), laser merge module containing 491, 561 and 642 laser lines (Spectral Applied Research) and an HQ2 cooled CCD camera (Roper Scientific). All hardware was controlled via Metamorph acquisition software (MDS Analytical Technologies). Traction force data was obtained at 37°C in a perfusion chamber (Warner Instruments) using a 60×1.2 NA Plan Apo WI objective (Nikon). Cells were maintained in culture media supplemented with 10 mM HEPES and 30 µl/ml Oxyrase (Oxyrase, Inc.).

### Actin flow speed measurements

Cells were transfected with GFP-actin and plated on a 2.8 kPa PAA gel for live imaging. Images were collected at 30 second intervals for 30 minutes for each cell. Fiducial marks within the lamellar actin were identified and tracked over time using custom software developed by the Danuser Lab (Harvard University) as previously described [26]. Briefly, local intensity maxima were identified and tracked using time-integrated cross-correlations. Templates for the object size for the correlations were adaptively chosen as 1.6×1.6 µm. Approximately 1000–3000 flow vectors were generated for each image. Flow vectors were chosen from regions exhibiting coherent flow, and the means were calculated for each condition by averaging the magnitudes of the flow vectors from 5–7 suitable regions for each cell, for two cells per condition.

### Displacement analysis and force reconstruction

Methods for traction force microscopy have been previously described [22], [23], [25]. Briefly, images of fluorescent beads embedded in the PAA gel were aligned to compensate for experimental drift and the bead displacement field was calculated between pairs of images by comparing the unstrained bead images obtained after the cell had been removed to images obtained with an attached cell. Displacement fields were calculated using Particle Imaging Velocimetry (PIV) software in MATLAB (available at http://www.oceanwave.jp/softwares/mpiv/), using the Minimum Quadratic Differences (MQD) algorithm which calculates the shift necessary to produce the minimum cross-correlation coefficient between a small region of the experiment image and the reference image. Displacement vectors were filtered and interpolated using the kriging interpolation method. We used a displacement grid size of 0.86 µm for these measurements. From the displacement data, Fourier transform traction cytometry (FTTC) [27] was then used to estimate traction stress and force at focal adhesions [23]. Traction stresses were reconstructed with zeroth-order regularization, which has been shown to yield traction force measurements consistent with the boundary element method [23]. Regularization parameters remained constant for all data sets directly compared.

The total traction force exerted by cells was computed by summing the magnitudes of the traction stress vectors under and near the cell of interest, subtracting the background value (taken from a region outside of the cell – typically around 20 Pa) and multiplying by the area covered by the chosen traction stress vectors.

### Focal Adhesion Dynamics

Focal adhesion lengths and lifetimes were found by analyzing time-lapse images of focal adhesions by hand in order to measure the focal adhesion length as a function of time. Focal adhesion lifetimes were determined as the total amount of time a focal adhesion was present in the time-lapse movie. Focal adhesion elongation and disassembly rate were computed by dividing the change in focal adhesion length during assembly and disassembly. Forces at individual focal adhesions were measured by computing the stress at the focal adhesion by interpolating traction stress vectors using a Gaussian weight function and converting the peak stress measured in the adhesion into force by multiplying by the area of the stress footprint at the adhesion [25].

## Supporting Information

Video S1 **Time-lapse video of a wild type U2OS cell showing actin, paxillin, and heatmap of traction stress magnitude.** Time is in min:sec.
(MP4)

Video S2 **Time-lapse video of a U2OS cell in 2 µM Y-27632 showing actin, paxillin, and heatmap of traction stress magnitude.** Time is in min:sec.
(MP4)

Video S3 **Time-lapse video of a U2OS cell in 5 µM Y-27632 showing actin, paxillin, and heatmap of traction stress magnitude.** Time is in min:sec.
(MP4)

Video S4 **Time-lapse video of a U2OS cell in 10 µM Y-27632 showing actin, paxillin, and heatmap of traction stress magnitude.** Time is in min:sec.
(MP4)

Video S5 **Time-lapse video of a U2OS cell over-expressing α-actinin showing α-actinin, paxillin, and heatmap of traction stress magnitude.** Time is in min:sec.
(MP4)

Video S6 **Time-lapse video of a U2OS cell over-expressing constitutively active (CA) Dia1 showing paxillin and heatmap of traction stress magnitude.** Time is in min:sec.
(MP4)
